# Topical sterosomes-based nanocarrier of miconazole for the management of cutaneous candidiasis

**DOI:** 10.1371/journal.pone.0353060

**Published:** 2026-07-10

**Authors:** Maha Alsunbul, Randa Mohammed Zaki, Ghaida N. Alnuwaybit, Fatimah A. Aljunayh, Mohd Nazam Ansari, Najeeb Ur Rehman, Abubaker M. Hamad, Fatma I. Abo El-Ela, Ehssan Moglad, Mayada Said

**Affiliations:** 1 Department of Pharmaceutical Sciences, College of Pharmacy, Princess Nourah bint Abdulrahman University, Riyadh, Saudi Arabia; 2 Department of Pharmaceutics, College of Pharmacy, Prince Sattam Bin Abdulaziz University, Al-Kharj, Saudi Arabia; 3 Department of Pharmaceutics and Industrial Pharmacy, Faculty of Pharmacy, Beni-Suef University, Beni-Suef, Egypt; 4 Department of Pharmacology and Toxicology, College of Pharmacy, Prince Sattam Bin Abdulaziz University, Al-Kharj, Saudi Arabia; 5 Department of Basic Sciences, Al-Rayan National College of Nursing, Al-Rayan National Colleges, Al Madinah Al Munawarah, Saudi Arabia; 6 Department of pharmacology, Faculty of Veterinary Medicine, Beni-Suef University, Beni-Suef, Egypt; 7 Department of Pharmaceutics and Industrial Pharmacy, Faculty of Pharmacy, Cairo University, Cairo, Egypt; Laurentian University, CANADA

## Abstract

**Background:**

Miconazole (MN) is widely used to treat superficial fungal infections; however, limited skin penetration and short residence time restrict its therapeutic efficacy. This study aimed to develop and statistically optimize MN-loaded sterosomes (STEs) to enhance topical antifungal activity.

**Methods:**

A central composite rotatable design (CCRD) was applied using Design-Expert® software to study the effects of cholesterol amount (mg) and sonication time (min) on vesicle size (VS), zeta potential (ZP), and entrapment efficiency (EE%). Vesicle morphology was characterized by transmission electron microscopy (TEM), and drug entrapment was confirmed using X-ray diffraction (XRD). The optimized formulation was incorporated into a hydroxypropyl methylcellulose (HPMC) gel and evaluated for *in vitro* release and *in-vivo* antifungal efficacy in a Wistar albino rats cutaneous candidiasis model (n = 6) following topical administration of optimized MN-loaded sterosome gel 1% w/w for ten days.

**Results:**

The optimized formulation showed a desirability of 0.63 and consisted of 140.86 mg cholesterol and 8.99 min sonication time. It demonstrated vesicle size: 498.54 ± 6.12 nm, zeta potential: 40.82 ± 1.24 mV, entrapment efficiency: 77.41 ± 1.43%. MN release from STEs was significantly higher than the drug suspension. TEM images showed spherical non-aggregated vesicles. XRD patterns indicated successful MN entrapment. *In-vivo*, MN-STE gel produced significantly greater antifungal activity than commercial Daktarin® cream at a lower dose, which was consistent with histopathological improvement.

**Conclusion:**

MN-loaded sterosomes enhanced drug entrapment, release, and antifungal efficacy while enabling dose reduction, representing a promising carrier for topical miconazole delivery.

## 1. Introduction

Fungal infections are increasing globally and present a growing challenge to healthcare systems. This rise is largely associated with expansion of immunocompromised populations resulting from intensive chemotherapy, use of immunosuppressive drugs, or underlying conditions such as human immunodeficiency virus (HIV) infection [1]. Secondary fungal infections have also been reported in patients with severe coronavirus disease 2019 (COVID-19) or post-COVID-19 syndromes, most commonly invasive candidiasis and aspergillosis [2].

The skin hosts a diverse microbiota that contributes to maintaining the balance between health and disease. It functions as the first line of defense against external pathogens through its physical and biological barrier properties. The outermost stratum corneum plays a major role in restricting penetration of microorganisms and exogenous substances owing to its unique structure and physicochemical characteristics [3,4]. The cutaneous microbiome comprises bacteria, archaea, viruses, and fungi; among them, species of Candida are frequent commensals and may become opportunistic pathogens under favorable conditions [5].

Candidiasis is the most prevalent fungal infection and includes both superficial and deep forms. Cutaneous candidiasis is characterized by inflammatory lesions of the skin, nails, and mucous membranes, commonly caused by Candida albicans when the host–pathogen equilibrium is disturbed [6,7]. Clinical presentation varies by site and may include erythema, pruritus, swelling, fissures, or vesicles; mucosal infection may present with white plaques [2].

Topical antifungal therapy is generally preferred for superficial infections because it enables local delivery of the drug to the affected area while minimizing systemic exposure and associated adverse effects [8,9]. However, conventional topical formulations are often limited by inadequate dermal penetration, suboptimal residence time, and inconsistent drug levels at the site of infection. Formulation-related shortcomings such as greasiness, irritation, and drug instability may further reduce patient adherence [9].

Nanotechnology-based drug delivery systems have therefore been investigated to improve skin penetration and sustain drug release. Systems such as liposomes, solid lipid nanoparticles, polymeric nanoparticles, and dendrimers have been explored for antifungal therapy [9,10]. Liposomes are particularly attractive due to their biocompatibility and ability to encapsulate both hydrophilic and lipophilic compounds, protect drugs from degradation, and modulate release profiles [11]; however, conventional phospholipid liposomes may exhibit limited stability during storage and application [12], as well as suboptimal penetration across the highly organized lipid matrix of the stratum corneum [13,14]. Niosomes, composed of non-ionic surfactants, offer improved stability but may still exhibit limited permeation efficiency [15]. Ethosomes, which contain high concentrations of ethanol, enhance skin permeation by disrupting and fluidizing stratum corneum lipids; however, their high alcohol content may lead to irritation and reduced formulation stability [16].

Sterosomes are sterol-rich, non-phospholipid vesicular systems composed of single-chain amphiphiles (e.g., stearylamine or palmitic acid) and high cholesterol content [12,17]. Unlike conventional liposomes, sterosomes form liquid-ordered bilayers characterized by tighter molecular packing, enhanced rigidity, and superior physicochemical stability. The high cholesterol content plays a crucial role in modulating membrane organization, reducing bilayer permeability, and improving drug retention, while also promoting interaction with skin lipid domains [17,18]. These structural characteristics distinguish sterosomes from other vesicular carriers and contribute to their improved performance in topical delivery systems. Sterosomes have been investigated as carriers in diverse applications, including delivery of vancomycin against Staphylococcus aureus and methicillin-resistant Staphylococcus aureus infections, metformin for cancer therapy, and osteoinductive oxysterol combinations [17,19,20].

Miconazole nitrate (MN) is a broad-spectrum antifungal agent commonly used in the treatment of candidiasis, dermatophytosis, and other superficial mycoses. Its primary mechanism involves inhibition of ergosterol synthesis, leading to disruption of fungal cell membranes; additional effects include interference with oxidative enzymes [2,8]. Although MN is available in multiple topical dosage forms, therapeutic performance may be limited by low skin permeability and local irritation [21,22]. Novel formulation strategies, including nanosystems, have been investigated to improve its cutaneous delivery.

Therefore, the objective of this study was to develop and optimize miconazole-loaded sterosomes for topical application in cutaneous candidiasis. The working hypothesis was that sterosome-based vesicles could enhance entrapment and provide improved drug release characteristics compared with conventional suspension. The study focused on formulation optimization and preliminary *in vitro* and *in vivo* evaluation; comprehensive bioavailability, allergenicity, toxicity, and long-term stability assessments were beyond the scope of this work and should be addressed in future studies.

## 2. Materials and methods

### 2.1 Materials

Miconazole nitrate, stearylamine, cholesterol, methanol, and chloroform were purchased from Sigma-Aldrich (St. Louis, MO, USA). Hydroxypropyl methylcellulose (HPMC; grade K4M) were all purchased from Sigma-Aldrich (St. Louis, MO, USA). Cellulose membrane (Mol. Wt. cut off = 12,000 Da). All other chemicals and reagents were of analytical grade and used as received.

#### Microorganisms and maintenance.

The *Candida albicans* strain (ATCC 10231 was used for the induction of cutaneous candidiasis in the rat model. The identity of the strain was verified through biochemical profiling and typical colony morphology on Sabouraud Dextrose Agar (SDA). The microorganism was maintained through periodic subculturing on SDA at 37°C. For experimental use, a standardized inoculum was prepared by suspending the 24-hour colonies in sterile phosphate-buffered saline (PBS) to reach a final concentration of **10**^**6**^ **CFU/mL**, as verified by hemocytometer counting and plate-count methods.

### 2.2 Statistical design of MN-loaded STEs

A central composite rotatable design (CCRD) was employed to investigate the effect of formulation variables on entrapment efficiency (EE%) (Y1), vesicle size (VS) (Y2), and zeta potential (ZP) (Y3). Design-Expert® software (Version 7, Stat-Ease, Minneapolis, MN, USA) was used to construct the experimental design.

Two independent variables were evaluated: cholesterol amount (X1; 100–300 mg) and sonication time (X2; 5–10 min), resulting in 13 experimental runs. Drug amount and stearyl amine were kept constant at 100 mg/10 mL. The design variables and responses are summarized in Table 1.

**Table 1:** Central composite rotatable design for optimization of MN-loaded STEs.

Independent variables	Levels	
	Low	High
Cholesterol amount (mg) (X1)	100	300
Sonication time (min) (X2)	5	10
**Dependent values (Responses)**	**Desirability**	
EE% (Y1)	Maximize	
Vesicles size (Y2)	Minimize	
Zeta potential (Y3)	Maximize	

### 2.3 Preparation of MN-loaded STEs

MN-loaded sterosomes were prepared using the ethanol-injection method [23]. Briefly, miconazole nitrate (100 mg), stearylamine (100 mg), and the required amount of cholesterol were dissolved in 8 mL of organic solvent mixture (chloroform: methanol, 3:1 v/v). The organic phase was injected dropwise into 10 mL of pre-heated aqueous phase (80 °C) under magnetic stirring (1000 rpm) for 1 hour to obtain a milky dispersion. The dispersion was probe-sonicated for the predetermined sonication time according to design settings.

#### Preparation of drug suspension for comparison.

A plain miconazole suspension containing an equivalent drug concentration was prepared by dispersing MN in distilled water containing 0.5% w/v Tween 80 by vortex mixing for 5 minutes and sonication for 2 minutes.

### 2.4 Characterization of formulations

#### 2.4.1 Entrapment efficiency (EE%).

MN-loaded STEs were separated from unentrapped drug by centrifugation (SIGMA 3–30 K, Sigma, Steinheim, Germany) at 17,000 rpm for 60 min at 4 °C [24,25]. The supernatant was diluted and MN concentration was quantified at 232 nm using UV-visible spectrophotometer (Shimadzu UV-1800, Kyoto, Japan). The method was linear over 6–30 µg/mL (R² = 0.9999).

The following formula was used to determine the EE%:


$$\begin{array}{c} \text{EE}\%=(\text{TD}-\text{FD})/\text{TD}\times \text{100} \end{array}$$

where TD is total drug and FD is free drug.

#### 2.4.2 Vesicle size, polydispersity index, and zeta potential.

The vesicle size (VS), polydispersity index (PDI), and zeta potential (ZP) were measured using a Zeta sizer Nano-ZS (Malvern Instruments, Worcestershire, UK) at 90° to the incident light beam. Samples were diluted 1:100 with distilled water prior to measurement and analyzed at 25 °C [25,26]. Each sample was measured in triplicate and reported as mean ± standard deviation.

### 2.5 Statistical analysis, optimization, and validation

Analysis of variance (ANOVA) was conducted using Design-Expert® software to determine the statistical significance of main and interaction effects. A desirability function approach was employed to maximize EE% and ZP and minimize VS. The optimized formulation predicted by software was prepared experimentally, and % error between predicted and observed values was calculated as: (expected values – actual findings)/(expected values) x 100 [27].

### 2.6 Evaluation of optimized formulation

#### 2.6.1 Transmission electron microscopy (TEM).

Morphology of optimized STEs was examined by TEM (JEOL JEM-1010, Tokyo, Japan). Samples were diluted, placed on carbon-coated copper grids, negatively stained with 2% w/v phosphotungstic acid, air-dried for 5 min, and imaged at 80 kV [28]. Both single-particle and field-view images were captured to assess particle distribution.

#### 2.6.2 *In-vitro* release study.

The membrane diffusion method was used to compare miconazole release in suspension and the optimized MN-loaded STEs. Firstly, the semi-permeable cellulose membrane was soaked for 24hr in phosphate buffer at room temperature. Then, the miconazole suspension and MN-loaded STEs were placed in the membrane. The receptor media was 300 mL phosphate buffer (pH 7.4) which was kept at 37 ± 0.5° C. Sink conditions were maintained by using phosphate buffer (pH 7.4) containing 1% Tween 80, which significantly enhanced the solubility of miconazole nitrate, ensuring that the release medium could dissolve at least three times the total amount of drug released. During the study period (4 hrs.) a 5 mL sample was withdrawn every 30 minutes from the receptor media to assess the amount of miconazole released which was immediately refilled with an equivalent amount of freshly prepared buffer to keep the sink conditions. Finally, a 0.20 μm nylon membrane filter was used to filter the withdrawn samples. Then, a UV\Vis spectrophotometer was used to determine the concentration of MN in the filtered samples after appropriate dilution [17]. The concentration of MN released at different time points was calculated applying the following equation [29]:


$$\begin{array}{c} Qn=\frac{CnxVr+\sum_{i=\text{1}}^{n-\text{1}}CixVs}{initialdrugcontent} \end{array}$$

Qn: Total percentage of MN that has been released

Cn: MN concentration in the receotor media at sample n.

Vs: The sample volume

Vr: The receptor medium volume

$\sum_{i=\text{1}}^{n-\text{1}}Ci$: The sum of the previously established concentrations

Plotting the fraction of MN released (Qn) at several time points vs. the relevant time was done in order to determine the release profile of the optimal MN-loaded STEs formula in comparison to the drug suspension. Release experiments were conducted in triplicate.

#### 2.6.3 Thermogravimetric analysis (TGA).

Thermogravimetric analysis was carried out using (NETZSCH, TG209F1 libra). Samples analyzed included:

(i) optimized formulation,
(ii) physical mixture of components,
(iii) pure MN.

Heating was performed in three steps: first step of decomposition within the temperature ranges from RT–100 °C, second step within the temperature range 100–250 °C. The subsequent steps (250–700 °C) [30].

#### 2.6.4 X-ray diffraction (XRD).

X-ray diffraction patterns were recorded for pure MN and the optimum MN loaded STEs formulation using Ultima IV Diffractometer (Rigaku Inc., Tokyo, Japan) over 20 range 0–60° at 10°/min [24]. Samples were gently dried, ground, and mounted on glass sample holders.

#### 2.6.5 Differential scanning calorimetry (DSC).

The pure MN, stearyl amine, cholesterol and their physical mixture were subjected to DSC analysis. A differential scanning calorimeter (DSC N-650; Scinco, Italy) was used to conduct DSC research. Approximately 5 mg of material was placed in its aluminum pan, and it was heated to 300° C at a rate of 10° C/min in a dry nitrogen atmosphere [30].

#### 2.6.6 *In-vitro* antifungal activity.

Antifungal activity was evaluated using agar-well diffusion against *Candida albicans* (10⁶ CFU/mL). Wells (8 mm) were filled with 100 μL of formulation or control. Plates were incubated at 37 °C for 24 h. Inhibition zones were measured in triplicate independent experiments and results reported as mean ± SD [2].

### 2.7 Preparation of topical MN-loaded STE gel

Hydroxypropyl methylcellulose ether (HPMC) (2.5% w/w) was gradually dispersed into the optimized MN-loaded STE dispersion under magnetic stirring at 1500 rpm for 30 minutes until a homogeneous gel formed.

### 2.8 *In-vivo* antifungal activity

The optimized MN-loaded STEs Gel’s *in-vivo* antifungal activity was tested using the methodology described by Qushawy *et al.* [8]. All animal experiments were strictly reviewed and approved by the Standing Committee of Bioethics Research (SCBR), Deanship of Scientific Research, Prince Sattam Bin Abdulaziz University (PSAU) (Approval No.: SCBR-332/2024) and were performed in strict compliance with the ARRIVE guidelines for reporting animal research.

#### 2.8.1 Animals and immunosuppression.

Twenty-Four Adult Male, 8–10-week-old Wistar albino rats (200–220 g) were utilized for this study. Animals were acclimatized for one week under standard housing conditions. Immunosuppression was induced using methylprednisolone (5 mg/kg, i.p.) once daily for three days prior to infection based on previously published *in vivo* antifungal protocols employing comparable rat models [31]. The dosage for the optimized sterosome gel was set at 1% w/w, representing a 50% dose reduction compared to the commercial 2% Daktarin® cream. This dose-sparing approach was adopted to investigate the capability of the vesicular system to enhance drug delivery. The safety of the formulation was confirmed through a pre-experimental skin tolerance test, which revealed no clinical or histological signs of inflammatory signs or irritation [31].

#### 2.8.2 Preparation of the fungal strain.

*Candida albicans* was cultured on Sabouraud dextrose agar (SDA) plates for 48 h at 30 °C. Colonies were harvested, washed twice with sterile physiological saline, and suspended in saline containing 0.1% Tween 80. The suspension was adjusted to obtain a final concentration of 10⁷ CFU/mL [32].

#### 2.8.3 Induction of fungal infection.

A 4 cm² area on each rat’s dorsal surface was shaved and gently cleaned with saline. An intradermal injection of 100 μL *Candida albicans* suspension (10⁷ CFU/mL) was administered into the center of the shaved area. The injected area was lightly massaged to distribute the inoculum evenly. Infection was confirmed visually after 72 h based on erythema, edema, and lesion development [32].

#### 2.8.4 Experimental design.

Animals were randomized into four groups (n = 6 per group):

Group 1: Negative control (uninfected, untreated)
Group 2: Positive control (infected, untreated)
Group 3: Infected + optimized MN-loaded sterosome gel (1% MN, topical)
Group 4: Infected + Daktarin® cream (2% MN, topical)

Treatments were administered once daily for 10 days.

The marketed Daktarin® cream was applied at 2% w/w, its labeled commercial strength, whereas the optimized sterosome gel was applied at 1% w/w to explore whether the vesicular system enables dose reduction while maintaining antifungal activity. This study therefore represents a dose-sparing exploratory comparison, not a strictly dose-equivalent bioequivalence study.

#### 2.8.5 Clinical evaluation.

Animals were monitored daily throughout treatment for local and systemic signs of infection. The infected skin area was examined for:

Erythema, edema, scaling or maceration, pustules or abscesses, and presence of white patches or plaques. Clinical improvement or persistence of lesions was documented photographically.

#### 2.8.6 Anesthesia, analgesia, and efforts to alleviate suffering.

To minimize pain and psychological stress during the induction of cutaneous candidiasis and subsequent clinical scoring, rats were deeply anesthetized using a combined intraperitoneal (IP) injection of Ketamine hydrochloride (90 mg/kg) and Xylazine (10 mg/kg). Animals were closely monitored during the anesthetic window for the loss of the pedal reflex to ensure full surgical-plane anesthesia before any procedure.

Strict refine-and-minimize protocols were executed to alleviate animal suffering throughout the 10-day treatment timeline. Animals were housed in a climate-controlled room (22 °C, 55% relative humidity) under a standard 12-hour light/dark cycle with free access to sterilized water and a standard pellet diet. Cages were lined with clean, autoclaved bedding and changed regularly to avoid secondary infections. Welfare assessments (evaluating weight loss, mobility, grooming habits, and localized skin inflammation) were conducted twice daily by an experienced veterinarian.

#### 2.8.7 Methods of humane sacrifice (Euthanasia).

At the end of the 10-day treatment protocol, humane euthanasia was performed to harvest skin biopsies for histopathological profiling. To ensure a pain-free, non-stressful transition that would not biochemically or structurally interfere with the evaluated topical treatment, animals were subjected to an anesthetic overdose via an IP injection of the Ketamine/Xylazine cocktail at triple the surgical dose, followed immediately by cervical dislocation to confirm death. This method guarantees immediate cardiac and respiratory arrest without causing systemic cellular damage, thereby preserving the integrity of the epidermal architecture for subsequent histopathological validation.

#### 2.8.8 Histopathological examination.

Skin tissue samples with a thickness of roughly 1 cm were obtained from all the animals utilized in this study after the treatment period. These tissue samples were processed using an automatic tissue processing machine (ASP300s, Leica Biosystems, IL, USA) after being promptly immersed in a sufficient volume of 10% formalin. Following tissue processing, these tissue samples were embedded in paraffin wax blocks and using a rotary microtome (SHUR/Cut 4500, TBS, NC, USA), sections of each tissue block with a thickness of 5 µm were created. Each sample was divided into two sections for staining; the first was stained using the general staining method of hematoxylin and eosin (H&E), and the second was stained using Masson trichrome (MT), a stain method specifically designed for connective tissue fibers [33]. For H&E processing, sections were deparaffinized and rehydrated using decreasing ethanol to water grades. If required, the fixed pigments were also removed. For ten minutes, the segments were submerged in hematoxylin (HX082464, MERK, Darmstadl, Germany) followed by washing under running water for five to ten minutes or less, or until parts turn “blue.” For ten minutes, stained in 1% eosin Y followed by one to five minutes washing under running water. Increasing ethanol was used to dehydrate, then clean and mount in DPX. To demonstrate connective tissue fibers (mostly collagen) using the MT technique, nuclei were stained with Weigert’s iron hematoxylin for ten minutes, then rinsed quickly with water, stained for five minutes in an acid fuchsin solution, differentiated for about five minutes in 1% phosphomolybdic acid, drained and counterstained with methyl blue, dehydrated, clear, and mounted in DPX [33].

### 2.9 Statistical analysis

All data are presented as mean ± standard deviation. Statistical comparisons were performed using one-way ANOVA. Differences were considered statistically significant at p < 0.05.

## 3. Results and discussion

### 3.1 Evaluation of MN-loaded STE formulations

#### 3.1.1 Entrapment efficiency (EE%).

**Fig 1:**
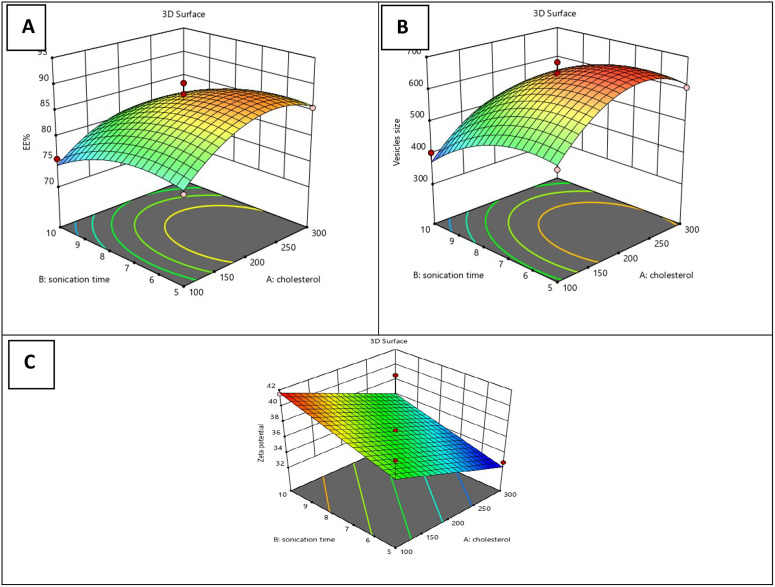
3D Response surface plot for the effect of independent variables on the dependent responses, (A) EE%, (B) Vesicles size, and (C) Zeta potential.

The entrapment efficiency (EE%) of MN-loaded sterosome formulations ranged from 73.5% to 90.3% (Table 2). This indicates efficient incorporation of MN into the sterosomal vesicles. The effects of cholesterol amount (X1) and sonication time (X2) on EE% are shown in Fig 1A and 1B The EE% data showed a good fit (p-value = 0.0010) to the quadratic model, whereas the lack of fit (p-value = 0.5660) wasn’t significant as shown in Table 4. The model considered valid when the difference between the adjusted and projected R^2^ values was less than 0.2, as reported by [34,35]. According to Tables 3, the model’s adequate precision of 9.8291 suggests that it might function effectively within the designated area [36].

**Table 2:** Composition of different formulations according to Central Composite Design.

Formula	Independent Variables		Dependent Variables			
	Cholesterol amount (mg) (X1)	Sonication time (min) (X2)	EE% (Y1)	Vesicle size (nm) (Y2)	Zeta potential (mv) (Y3)	PDI
STEMs1	200	7.5	85.2 ± 1.20	597 ± 5.43	37 ± 1.23	0.667
STEMs2	341.421	7.5	80.8 ± 0.93	520 ± 7.53	32.7 ± 1.56	0.621
STEMs3	300	10	80.3 ± 2.13	516 ± 4.56	38.6 ± 2.01	0.449
STEMs4	200	7.5	88.2 ± 2.04	653 ± 8.76	36.4 ± 1.11	0.638
STEMs5	58.5786	7.5	73.5 ± 0.84	368 ± 8.56	41.3 ± 0.94	0.667
STEMs6	200	7.5	90.3 ± 1.74	684 ± 9.65	35.2 ± 1.54	0.583
STEMs7	300	5	85.6 ± 1.96	607 ± 8.43	32.7 ± 2.02	0.587
STEMs8	200	7.5	85.7 ± 2.11	649 ± 10.5	35.5 ± 1.78	0.621
STEMs9	100	10	75.6 ± 1.98	402 ± 6.54	41.5 ± 1.19	0.532
STEMs10	200	7.5	86.2 ± 2.36	650 ± 8.23	35.8 ± 2.23	0.423
STEMs11	200	3.96447	86.5 ± 2.27	650.4 ± 10.38	33.2 ± 0.87	0.621
STEMs12	200	11.0355	75.1 ± 3.45	400.1 ± 9.45	39.2 ± 0.97	0.630
STEMs13	100	5	78.4 ± 3.21	496.3 ± 5.41	39.8 ± 1.47	0.476
Optimized formula	140.858	8.92				

**Table 3:** ANOVA for Central Composite Design of MN-loaded STEs.

Dependent Variable	Source	SS	Df	Mean Square	F value	p value	
**Y1**	**Model**	329.79	5	65.96	16.38	0.0010	Significant
	A-cholesterol	61.74	1	61.74	15.33	0.0058	
	B-sonication time	73.34	1	73.34	18.22	0.0037	
	AB	1.56	1	1.56	0.3881	0.5531	
	A²	155.97	1	155.97	38.74	0.0004	
	B²	58.91	1	58.91	14.63	0.0065	
	Lack of Fit	10.35	3	3.45	0.7744	0.5660	not significant
**Y2**	**Model**	1.379E + 05	5	27571.68	23.13	0.0003	Significant
	A-cholesterol	24162.67	1	24162.67	20.27	0.0028	
	B-sonication time	36352.55	1	36352.55	30.50	0.0009	
	AB	2.72	1	2.72	0.0023	0.9632	
	A²	64278.37	1	64278.37	53.93	0.0002	
	B²	21427.83	1	21427.83	17.98	0.0038	
	Lack of Fit	4425.36	3	1475.12	1.51	0.3415	not significant
**Y3**	**Model**	93.74	2	46.87	25.18	0.0001	Significant
	A-cholesterol	61.40	1	61.40	32.99	0.0002	
	B-sonication time	32.34	1	32.34	17.38	0.0019	
	Lack of Fit	16.53	6	2.75	5.28	0.0647	not significant

**Table 4:** The composition and validation of the optimized formula with its predicted responses according to Central Composite Design.

Responses	Predicted value	Experimental value	% Relative error
EE%	81.3735	77.410 ± 1.43	4.870
Vesicles size	524.031	498.542 ± 6.12	4.864
Zeta potential	39.6185	40.822 ± 1.24	3.037

The subsequent equation showed how the formulation variables affected EE%:


$$\begin{array}{c} \text{EE}\%=+\text{87}.\text{12}+\text{2}.\text{78 X1}-\text{3}.\text{03 X2}-\text{0}.\text{6250 X1X1}-\text{4}.\text{73}{\left(\text{X1}\right)}^{\text{2}}-\text{2}.\text{91}{\left(\text{X2}\right)}^{\text{2}} \end{array}$$

The cholesterol amount (X1) and sonication time (X2) have a significant impact on EE% with p-values of 0.0037 and 0.0037, respectively as revealed by the ANOVA analysis Table 3. As evident in the correlation equation, cholesterol amount showed a positive influence on EE% while sonication time had a negative effect. Where increasing the cholesterol concentration led to a significant increase in EE% which could be related to increasing the hydrophobicity of the bilayer structure that enhances the solubilization and entrapment of the lipophilic MN into the bilayer during vesicles formation. Also, the increase in cholesterol concentration increases the rigidity of the bilayer membrane which increases the rigidity of the membrane and decreases the drug leakage [17,27,37]. Conversely, the increase in the sonication time led to a significant decrease in entrapment efficiency which could be attributed to the reduced vesicle size of the STEs formulations with the subsequent reduced amount of the entrapped drug [29].

#### 3.1.2 Evaluation of vesicle size (VS), polydispersity index (PDI), and zeta potential (ZP).

**Fig 2:**
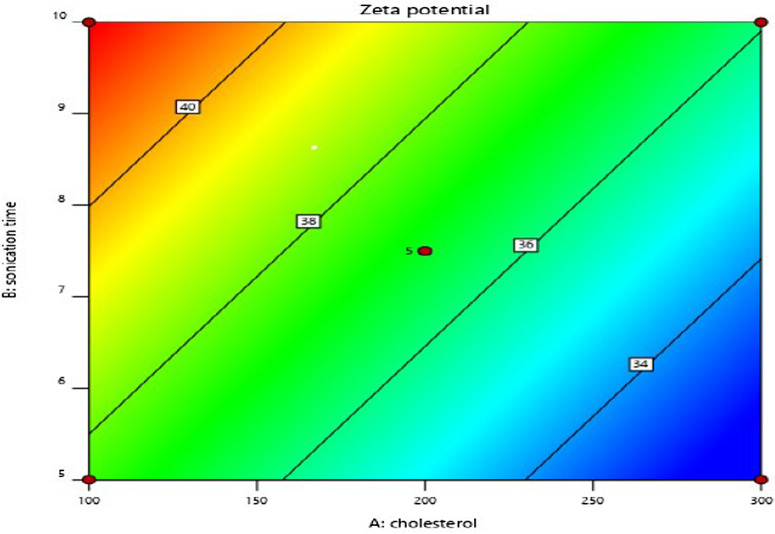
Contour plot for the effect of independent factors on different responses.

The vesicle size (VS) of various sterosomal formulations varied between 400.1 to 684 nm, as shown in Table 2. Increasing the sonication time (X2) and decreasing the cholesterol amount (X1) gives a smaller vesicle size in Figs 1B and 2B. The VS data was best fitted to the quadratic model (p-value = 0.0003) with a nonsignificant lack of fit (p-value = 0.3415) as shown in Table 3.

The effect of the studied factors on VS could be studied using the following correlation equation (2).


$$\begin{array}{c} \text{VS}=+\text{646}.\text{60}+\text{54}.\text{96 X1}-\text{67}.\text{41X2}+\text{0}.\text{8250X1X1}-\text{96}.\text{12 }{\left(\text{X1}\right)}^{\text{2}}-\text{55}.\text{50 }{\left(\text{X2}\right)}^{\text{2}} \end{array}$$

According to the ANOVA analysis in Table 3, cholesterol amount (X2) and sonication time (X1) have a significant impact on VS with p-values of 0.0028 and 0.0009, respectively. The increase in the cholesterol amount (X2) showed a negative impact on VS while sonication time (X1) presented a positive impact on VS as shown by the sign in the correlation equation (2).

Concerning the effect of sonication time (X1) on VS, longer sonication time (X2) produces smaller VS which may be associated with the pressure exerted by ultrasonic waves on the colloidal formulation, leading to a reduction in particle fraction and size [24,38].

Conversely the increase in cholesterol concentration increases the vesicle rigidity, which results in resistance to size reduction during the sonication [37].

The polydispersity index (PDI) values of the various formulations range between 0.423–0.667. The PDI refers to the range of sizes in lipidic nanocarrier systems and is used to characterize how ununiform a particle size distribution is. PDI is a highly monodisperse standard when the values become less than 0.05 while a PDI value higher than 0.7 represents a very broad particle size distribution. Furthermore, data that lies between these two extreme PDI values 0.05–0.7 are handled differently by different size distribution algorithms.

The zeta potential (ZP) represents the physical stability of the sterosomal formulations. Larger ZP value indicates higher repulsion forces between vesicles which leads to reduced aggregation and increased stability of the system. In Figs 1C and 2, high cholesterol amount leads to lower ZP and sonication time leads to high ZP. The model which best fits the ZP data was the linear model (p-value = 0.0001) where the lack of fit was non-significant (p-value = 0.0647).

Based on Table 3, cholesterol amount (X1) and sonication time (X2) have a significant impact on ZP with p-values of 0.0002 and 0.0019, respectively. Cholesterol amount (X1) showed a negative impact on ZP [39] while sonication time (X2) presented a positive impact on ZP as shown in the correlation equation (3). The decrease in zeta potential with the increase of cholesterol amount could be related to the neutralization of the positive charge of stearyl amine with the negative charge of cholesterol [40]. The increase in zeta potential with the increase in sonication time could be related to the breakdown of larger vesicles by the high energy of the ultrasonic waves into tiny discoid fragments that fold up and produce thermodynamically stable vesicles [41].


$$\begin{array}{c} \text{ZP }=+\text{36}.\text{84}-\text{2}.\text{77X1}+\text{2}.\text{01X2} \end{array}$$

### 3.2 Statistical analysis, optimization, and validation

**Fig 3:**
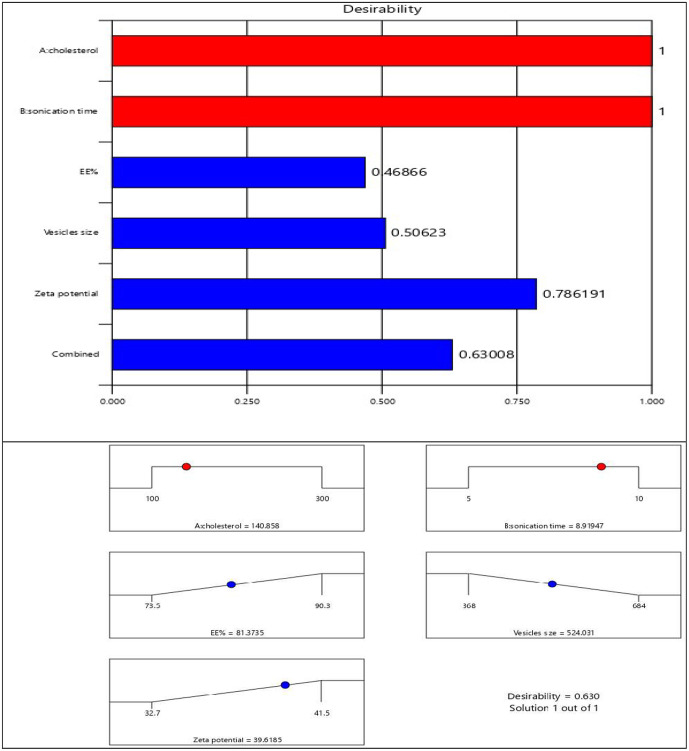
Desirability Bar graph and the expected responses of the optimized formula according to Central Composite Design.

**Fig 4:**
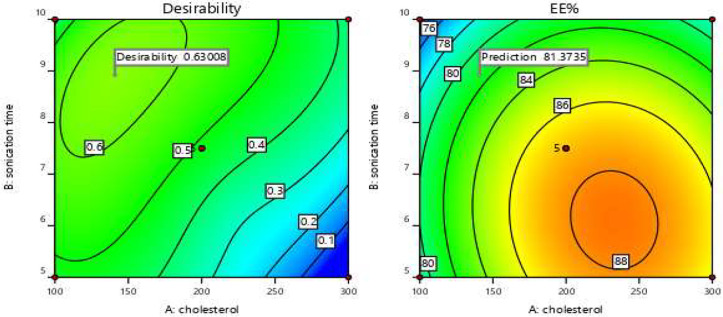
Contour graphs for the expected responses of the optimized formula with its desirability.

Design Expert® software (Ver. 7, Stat-Ease, Minneapolis, MN, USA) determined the best formula by minimizing VS while maximizing ZP, and EE%. The optimized sterosomal formulations have a desirability of 0.630 with EE%, VS, and ZP predicted values of 81.3735, 524.031 nm, and 39.6185 mv, respectively, as shown in Fig 3. It was composed of 140.858 mg cholesterol, 100 mg stearyl amine and subjected to 8.99 minutes sonication. The optimal formula was formulated and validated, as illustrated in Table 4, exhibiting a relative error of under 5% from the anticipated results generated by the Design Expert software, so affirming model fitness [24]. Fig 4 shows the contour graphs for the expected responses of the optimized formula with its desirability.

### 3.3 Evaluation of the Optimum MN loaded STEs

#### 3.3.1 Transmission electron microscopy (TEM).

**Fig 5:**
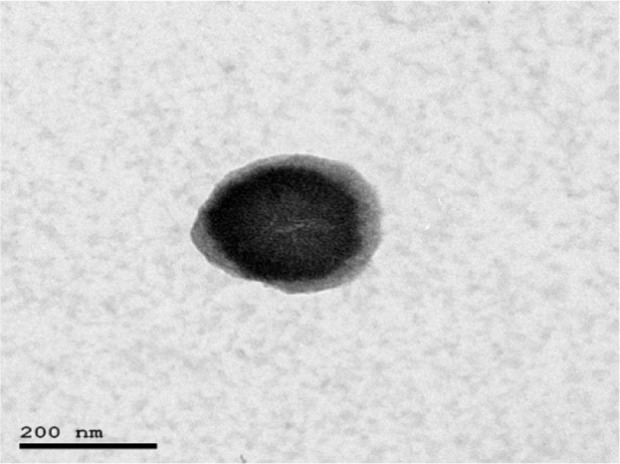
TEM image of the optimum MN loaded STEs formulation.

As seen in Fig 5, TEM imaging revealed reasonably spherical vesicles. No aggregates were seen, which could be explained by the surrounding STEs being repelled by the relatively high ZP on the vesicle surfaces [42].

#### 3.3.2 *In-vitro* drug release.

**Fig 6:**
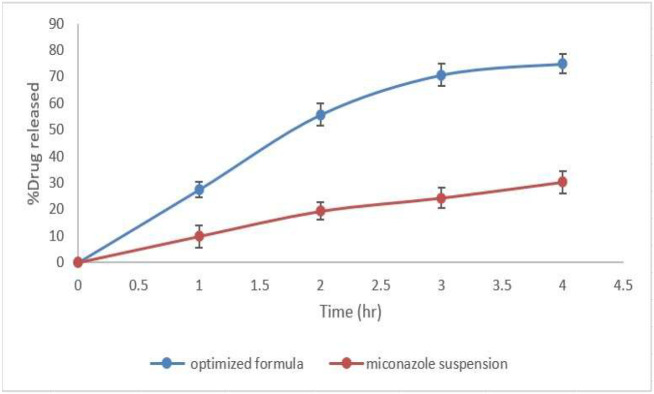
*In-vitro* release of MN loaded STEs versus MN suspension.

As revealed in Fig 6, the optimized MN-loaded STEs have a higher % drug release compared to miconazole suspension which may be related to the increased solubilization of MN insides the STEs besides the large surface area of STEs resulting from the low vesicles^,^ size [43].

Table 5 shows the release kinetics of optimized MN-loaded STEs compared to drug suspension.

**Table 5:** The release kinetics of optimized MN-loaded STEs compared to drug suspension.

Formula	Linear Regression Analysis Using Correlation Coefficient R_2_ According to			
	Zero	First	Second	Higuchi
Optimized MN-loaded STEs	0.931837	0.97767	0.976496	0.968392
Miconazole suspension	0.978792	0.990183	0.996237	0.967715

#### 3.3.3 Thermogravimetric analysis (TGA).

**Fig 7:**
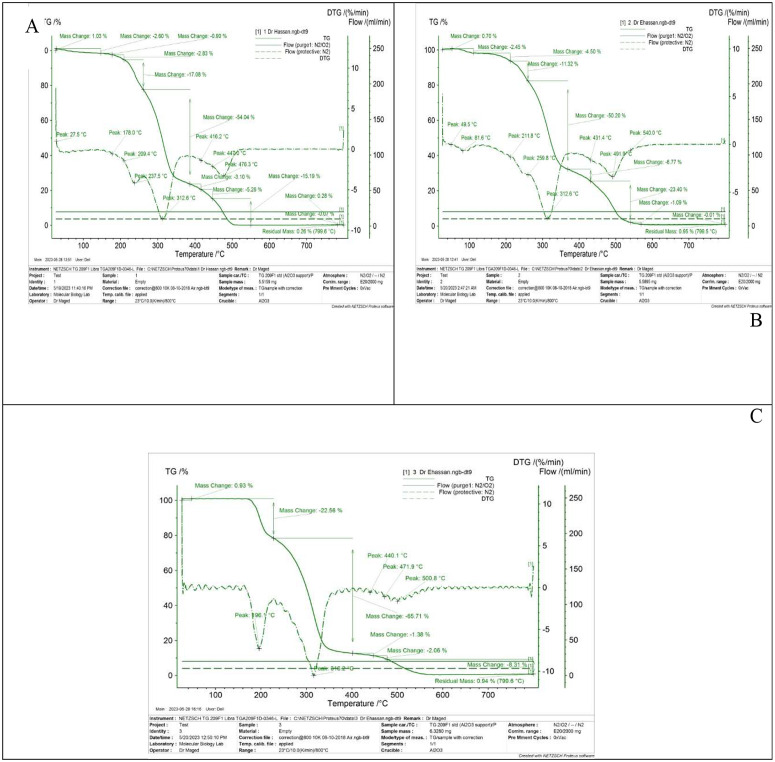
Thermogravimetric analysis of (A): optimized MN loaded STEs, (B): physical mixture of MN, cholesterol, and stearyl amine, (C): MN pure drug. A and B nearly the same (only using water as solvent increases the water content on turn the % weight loss in the samples).

**3.3.3.1 Sample (1) using H**_**2**_**O as Solvent:** Blending MN drug with cholesterol and stearyl amine significantly improves the thermal properties. The thermograms indicated a first step of decomposition within the temperature range from RT–100 °C, which corresponds to the removal of the hydrated water molecules with a mass loss of 1.03%. In the second stage within the temperature range 100–250 °C, the weight loss was found to be ~ 23.41 and this may be due to the decomposition of HCl molecules. The subsequent steps (250–700 °C) correspond to the complete decomposition of the blend and the overall weight loss amounts 99.74% (the residual mass 0.26) as shown in Fig 7A.

**3.3.3.2 Sample (2) where Physical mixing of the blend compositions:** a first step of decomposition within the temperature range RT–100 °C, which corresponds to the removal of the hydrated water molecules with a mass loss of 0.07%. In the second stage within the temperature range 100–250 °C, the weight loss was found to be ~ 18.27 and this may be due to the decomposition of HCl molecules. The subsequent steps (250–700 °C) correspond to the complete decomposition of the blend and the overall weight loss amounts 99.05% (the residual mass 0.95) as shown in Fig 7B.

**3.3.3.3 TGA of MN in sample (3):** The material shows an estimated mass loss of 23.49% within the temperature range of room temperature (RT) to 250 °C, likely due to the release of C₃H₃N₂ and HCl as gases. In the subsequent stages, between 250 °C and 700 °C, the remaining C₁₅H₁₀Cl₃O structure undergoes decomposition, resulting in a further mass loss of 74.02%. This complete breakdown produces gases such as CO, CO₂, NO, and NH₃, as illustrated in Fig 7C [30].

Sample (1) and (2) nearly the same (only using water as solvent increases the water content an on turn the % weight loss in the samples)

#### 3.3.4 X-ray diffraction study (XRD).

**Fig 8:**
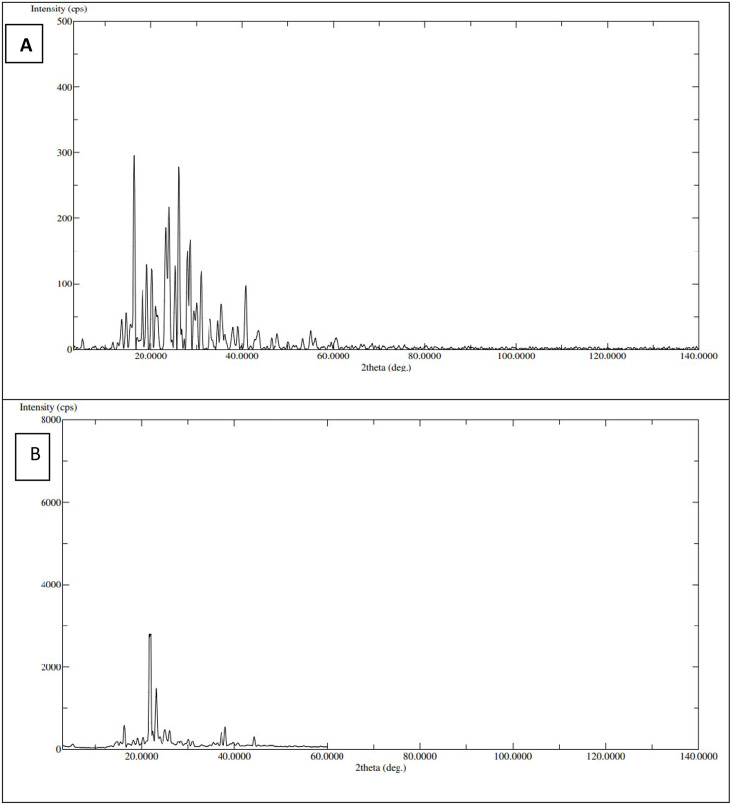
XRD patterns of A: pure MN and B: the optimum MN loaded STEs formulation.

Fig 8 displays the XRD spectra of pure MN and the optimized STEs formulation. The MN spectrum revealed distinctive, sharp peaks that indicated crystallinity (Fig 8A) [44]. While some MN peaks showed reduced intensity and the absence of others in the XRD spectra of the optimized MN loaded STEs (Fig 8B), this could be related to MN being entrapped inside STEs vesicles in an amorphous form [29,44].

#### 3.3.5 Differential scanning calorimetry (DSC).

**Fig 9:**
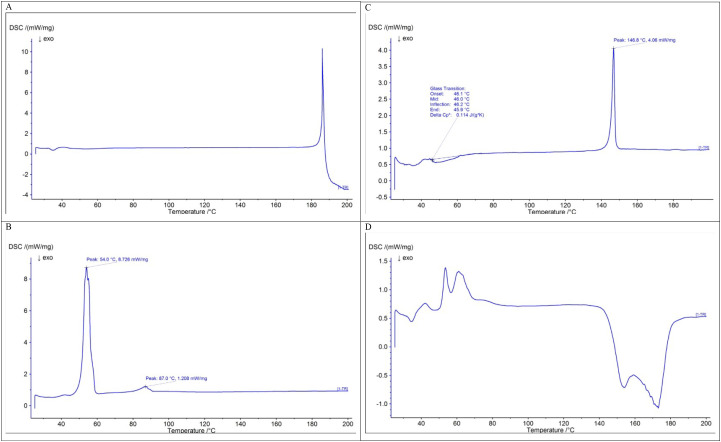
DSC thermograms of (A) pure MN, (B) stearylamine, (C) cholesterol and(D) physical mixture.

DSC was used to examine the physicochemical characteristics and thermal behavior of pure MN, stearyl amine, cholesterol and physical mixture (Fig 9). While FTIR is often utilized to detect specific hydrogen bonding, DSC was employed in this study as a robust alternative to confirm physicochemical compatibility by demonstrating the physical state transition and molecular dispersion of MN within the lipid matrix, thereby confirming the absence of new crystalline phases or chemical incompatibilities. DSC thermograms are helpful in identifying the nature or physical state of drug molecules both when they are loaded in nano formulations and when they are not, since the physical state of drug molecules in nanoformulations influences their release and solubility in external media [45]. MN’s particular melting point was confirmed by a prominent endothermic peak at about 186°C [46] (Fig 9A). DSC thermogram of stearylamine showed an endothermic peak at 54°C [47](Fig 9B) while DSC thermogram of cholesterol showed an endothermic peak at 146.8°C [46] (Fig 9C) [48]. DSC thermogram of the physical mixture (Fig 9D) showed disappearance of the characteristic peaks of MN and cholesterol and the appearance of a new exothermic peak while the intensity of stearyl amine peak was reduced. The disappearance of the melting peaks of MN and cholesterol could be related to that both components dissolve into molten lipids rather than melting as separate crystalline phases. A behavior also observed when drugs lose their endotherms after mixing with lipids that disrupt their crystalline structure [49, 50]. The new exothermic peak reflects formation of a reorganized mixed phase with a different heat capacity. This is similar to reports where new thermal events arise upon creation of new compound‑like structures in mixtures [51]. The reduced stearylamine peak intensity indicates partial distortion of its crystalline lattice. This is consistent with observations that interactions with other lipids weaken or suppress its melting transition [52,53].

#### 3.3.6 *In-vitro* antifungal activity.

**Fig 10:**
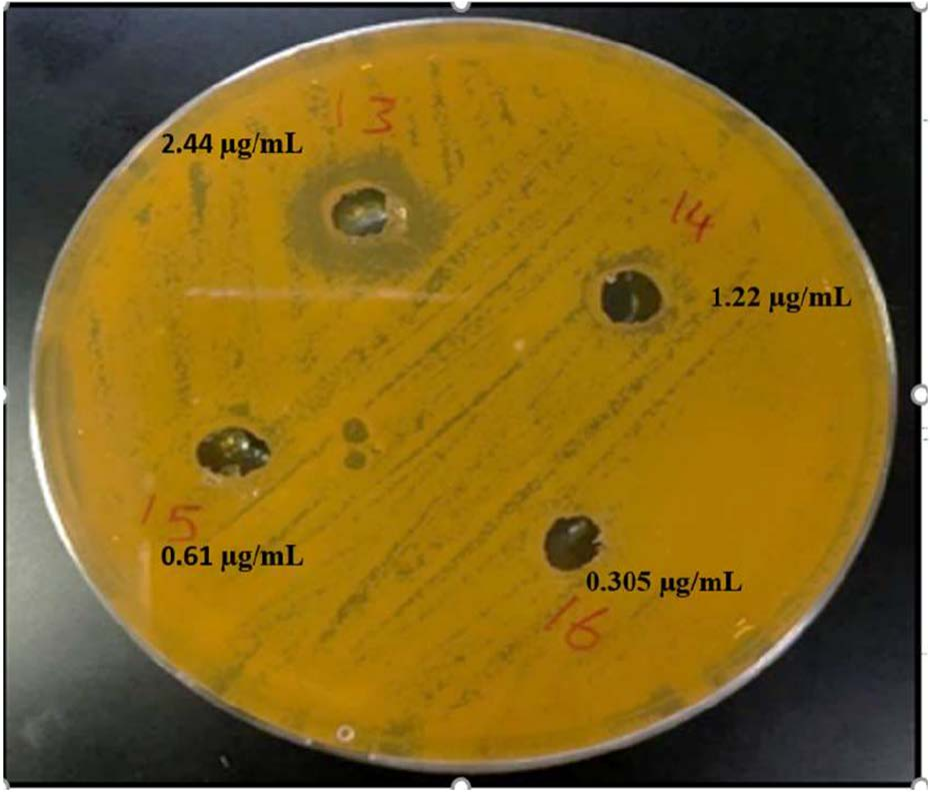
Inhibition zones of optimized MN formula with MIC of 1.22 µg/mL.

**Fig 11:**
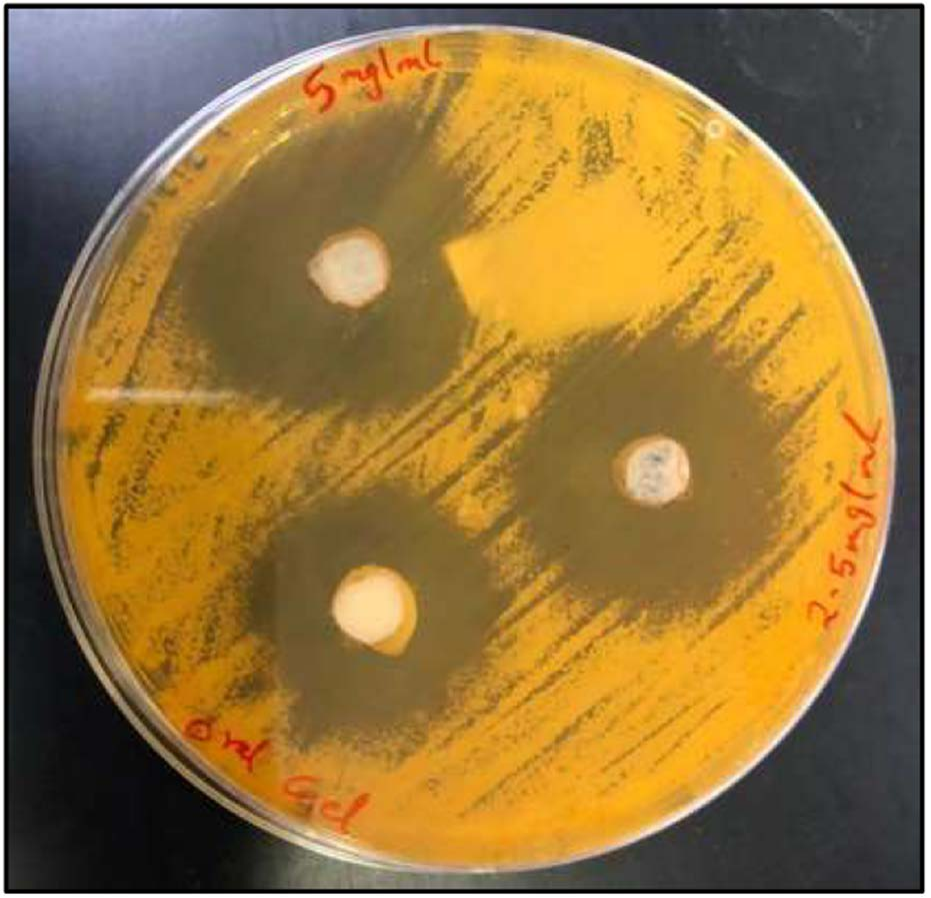
Inhibition zones of optimized MN formula (2.5 and 5 mg\ml) and commercial oral gel.

The optimized MN-loaded STEs showed remarkably high growth inhibition to *C. albicans* at concentrations ranging from 2312.5 µg/mL with MIC = 1.22 µg/mL as shown in Fig 10 and Table 6. However, at 2.5 and 5 mg\ml of the optimized MN-loaded STEs showed better inhibition to *C. albicans* as compared to 200 mg\ml of the commercial MN gel as shown in Fig 11 because one or both of the listed reasons: a) smaller particle size means a larger surface area and then higher intrinsic particle activity of the miconazole, b) smaller particle size enhances the MN diffusion within the SDA medium which increasing the inhibition zone [54].

**Table 6:** Inhibition zone in mm for antifungal activity for drug and optimized formula.

concentration (µg)	Standard drug (mm ± SD)	Optimized Formula (mm ± SD)
312.5	30.67 ± 0.58	32.33 ± 1.15
156.25	29.00 ± 1.00	30.33 ± 0.58
78.125	27.33 ± 0.58	28.67 ± 0.58
39.0625	23.33 ± 0.58	26.67 ± 0.58
19.53125	21.33 ± 0.58	24.33 ± 1.15
9.765625	19.67 ± 0.58	21.33 ± 0.58
4.8828125	18.67 ± 0.58	19.67 ± 0.58
2.44140625	17.67 ± 0.58	17.33 ± 0.58
1.220703125	14.67 ± 0.58	10.67 ± 0.58
0.6103515625	10.67 ± 0.58	–

#### 3.3.7 *In-vivo* antifungal activity.

**Fig 12:**
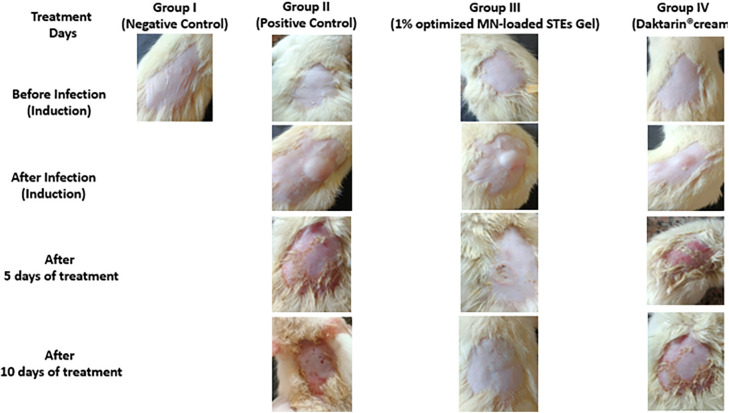
The skin of rats before and after a fungal infection was induced, as well as after receiving 1% optimal MN loaded STEs gel and 2% Commercial Daktarin® cream.

*Candida albicans* are frequently employed to evaluate the efficacy of an antifungal [55]. The contrast between the epidermis of the animal before and after induction is illustrated in (Fig 12).

A comparison of the skin was evaluated after 10 days of therapy with the commercially available cream (Daktarin® cream 2%) and 1% optimized MN loaded STEs gel. The epidermis structure of all animals was found to be normal antecedent to the induction of a cutaneous fungal infection that is not accompanied by any clinical symptoms, such as puffiness or edema, inflammation, scaling, and skin fissures.

Every day, the affected rodents were checked for any indications of infection. All the animals showed signs of infection, including skin discoloration, the day after the *Candida albicans* suspension was first administered. On the third day, it was scaling, and a clear erythema of the epidermis are more pronounced. The infection site exhibited evidence of discharge of the infectious agent after five days of treatment. Scales, exposing epidermis that was pale pink in color. After the 1% optimized MN loaded STEs gel treatment, the skin’s structure did not significantly change. After using the commercially available cream (Daktarin®) for 10 days, the edema and inflammation that accompanied the irritation decreased. The lesions continued to persist, despite the application of cream 2% (half dose was used). Conversely, the gel formula demonstrated the epidermis returned to its normal state after 10 days of treatment.

The MN loaded STEs gel treatment for a fungal infection showed a significant reduction in fungal infection as compared to other formulations and found to be more effective in preventing and treating the symptoms of infections caused by *Candida albicans*, according to the findings of an *in vivo* antifungal investigation. This might be explained by the drug’s improved ability to pierce deeper layers of the epidermis, which would increase its ability to stay there. Additionally, the presence of the edge activator has a synergistic effect on the retention of medications in the epidermis [56]. The improved therapeutic outcome observed despite the lower drug concentration suggests that sterosomes may enhance cutaneous deposition and local availability of miconazole. However, these findings should be interpreted cautiously, as formal dose-response, skin pharmacokinetic, and toxicity studies are needed to confirm true dose-sparing potential. Importantly, the enhanced skin permeation of miconazole observed in the present formulation can be attributed to the synergistic effects of vesicle composition and physicochemical properties. The incorporation of stearylamine imparts a positive surface charge to the sterosomes, which enhances electrostatic interaction with the negatively charged skin surface, thereby improving vesicle adhesion and facilitating drug deposition within the stratum corneum [57]. In addition, cationic lipids such as stearylamine have been reported to act as penetration enhancers by disrupting the ordered lipid structure of the stratum corneum and increasing lipid fluidity, which promotes drug diffusion across skin layers [57,58]. Furthermore, the high cholesterol content contributes to the formation of stable, ordered bilayers that can interact and partially fuse with skin lipids, enhancing drug partitioning into the skin [18]. The nanoscale size of the vesicles also facilitates their penetration through intercellular pathways [13]. Collectively, these factors—including cholesterol-mediated membrane organization, vesicle nanostructure, and the positive surface charge conferred by stearylamine-play a critical role in enhancing dermal delivery of miconazole.

#### 3.3.8 Histology.

**Fig 13:**
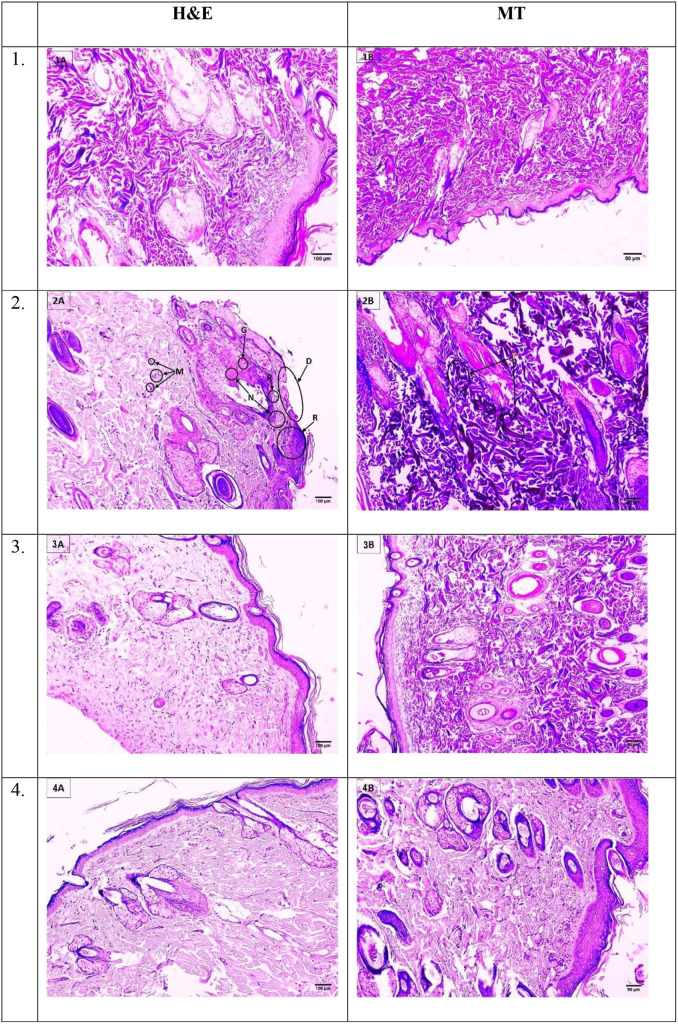
Histopathology small microphotograph results. 1A: Normal group, H&E stained section, showed normal general appearance. 1B: Normal group, MT stained section, showed normal connective tissue fibres distribution. 2A: Positive control group, H&E stained section, showed highly abnormal appearance including desquamation (D), microorganisms (M), degeneration (G), necrosis (N), and dysregulation (R). 2B: Positive control group, MT stained section, showed abnormal distribution and accumulation of collagen fibres (F). 3A: Infected + optimized MN-loaded sterosome gel (1% MN, topical) group, H&E stained section, showed almost normal general appearance. 3B: Infected + optimized MN-loaded sterosome gel (1% MN, topical) group, MT stained section, showed near to normal connective tissue fibres distribution. 4A: Infected + Daktarin® cream (2% MN, topical) group, H&E stained section, showed almost normal general appearance. 4B: Infected + Daktarin® cream (2% MN, topical) group, MT stained section, showed near to normal connective tissue fibres distribution.

The results showed that the effect of optimized MN-loaded STEs Gel treatment on histological studies. Photomicrographs of normal control group showed normal skin tissues with normal histological appearance, arrangement, and connective tissue fibers distribution and orientation (1A, H&E staining, 200x, scale 100µ), and also, normal distribution of collagen fibers (1B, MT staining, 300x, scale 50µ). Skin tissues of positive control group showed desquamation, highly infiltrating of the tissue sample with microorganisms (M), degeneration (G), necrosis (N) of large area, and dysregulation of tissues (R) (2A, H&E staining, 200x, scale 100µ) as well as abnormal distribution of collagen fibers (F) (2B, MT staining, 300x, scale 50µ). Skin tissues of animals treated with 1% optimized MN-loaded STEs Gel showed highly improvement and near to features of standard treatment due to good healing and restoring the tissue regulation and vivacity by presence of large numbers of fibroblasts to restore the normal fibers content of the dermis (3A, H&E, staining, 200x, scale 100µ). In addition, normal distribution of collagenic material in MT section was also seen (3B, MT, staining, 300x, scale 50µ). Animals treated with Daktarin®cream showed highly improvement and almost normal features, with the presence of little amount of the microorganism (4A, H&E staining, 200x, scale 100µ). In addition, normal distribution of collagenic material was also seen (4B, MT staining, 300x, scale 50µ) (Fig 13).

About the potent antifungal activity of the prepared formula; the integrated a mechanistic explanation of how the sterosome’s unique composition (high cholesterol and cationic charge) interacts with the stratum corneum to enhance drug penetration might be due to first: the Cationic-Electrostatic Interaction whereas the Skin’s surface is negatively charged (pH 4–6). Our prepared formula has + 40.82 mV charge acts as a “biological glue,” increasing the residence time on the stratum corneum. This prevents the drug from being wiped away or lost to desquamation, which is a major drawback of standard creams like Daktarin® [59]

About the Sterosome’s Rigidity & the Occlusive Effect; High cholesterol content (140.86 mg) in sterosomes makes the bilayer rigid but stable. Unlike traditional liposomes that might break down, sterosomes can form an occlusive film over the infected area. This increases skin hydration, which “opens up” the intercellular lipid pathways in the stratum corneum, allowing Miconazole to penetrate deeper into the dermis where Candida thrives [60].

While regards the Deformability & Size Factor; the formula size about is ~ 498 nm; While large for systemic entry, this size is ideal for follicular targeting. Fungi often hide in hair follicles and skin folds. The sterosome gel can “trap” the drug in these micro-reservoirs, creating a sustained-release depot. This explains why you saw better results than Daktarin® despite a lower dose.

So; The superior antifungal efficacy of the MN-STE gel over the commercial Daktarin® cream (p < 0.05) aligns with recent findings by [61]who demonstrated that lipid-based nanocarriers significantly bypass the skin’s barrier properties compared to conventional emulsions. While Daktarin® relies on a high drug concentration (2%) for passive diffusion, our sterosomal system (1%) utilizes active skin-vesicle interactions. This dose-sparing effect is consistent with the work of Okafor et al., [62] who showed that cationic sterosomes provide a 2.5-fold increase in dermal drug accumulation compared to standard topical products.

The histopathological improvement wasn’t just because the fungus died, but because the sterosomes reduced the local inflammatory response. Cationic surfactants can sometimes have mild intrinsic antimicrobial properties, which may have acted synergistically with the Miconazole [56].

### Skin biocompatibility and safety

The optimized MN-STE formulation exhibited a high positive zeta potential (+40.82 mV). While cationic vesicles can potentially cause skin irritation through interaction with skin lipids, the histopathological findings in this study confirmed the safety of the sterosomal gel. The skin sections treated with MN-STE showed a normal histological profile similar to the control group, with no evidence of inflammatory cell recruitment or epithelial disruption. This suggests that the sterosome matrix effectively encapsulates the components, mitigating potential irritancy while maintaining the benefits of cationic-enhanced skin adherence. SO; biocompatibility of Components: The sterosomes are primarily composed of cholesterol and stearylamine surfactant, used at concentrations optimized for stability rather than toxicity. *In Vivo* Observations: During the 10-day topical application in the Wistar rat model, no clinical signs of erythema, edema, or inflammation were observed in the treatment groups. Furthermore, our histopathological analysis (Fig 12) showed a restored epidermal structure with no signs of leucocyte infiltration or tissue damage, suggesting that the MN-STE gel was well-tolerated at the therapeutic dose.

*In Vivo* Efficacy and Histopathology act as “indirect” proof of dermatokinetics. Besides a formal tape-stripping kinetic study was not within the primary scope of this optimization phase, the enhanced therapeutic outcomes observed in the *in vivo* candidiasis model provide strong indirect evidence of superior skin retention and penetration.

Specifically, the significant reduction in fungal load and the complete restoration of the dermal-epidermal junction at a 50% lower dose compared to the commercial cream (1% vs. 2%) strongly suggests that the MN-loaded sterosomes facilitated deeper penetration into the infected dermis.

The vesicles around 500 nm are too large for systemic absorption but are perfectly sized to accumulate in hair follicles and skin furrows, which act as “drug reservoirs” (depots). The positive charge (+40.82 mV) creates an electrostatic attraction to the negative skin surface, preventing the “wash-off” effect common with standard gels. As, Vesicles in the range of 400–600 nm have been shown to preferentially accumulate in the pilosebaceous units, providing a localized depot for antifungal agents [63].

The high zeta potential of cationic sterosomes enhances the residence time of drugs in the stratum corneum by 3-fold compared to neutral formulations Omoteso etal..2025. Superior histopathological recovery at reduced doses is a hallmark of enhanced dermatokinetic penetration in lipid-based systems [64].

So; the improved antifungal activity of the MN-STE gel, despite its lower drug concentration, can be attributed to the unique dermatokinetic profile of sterosomes. The high cholesterol content provides structural integrity, while the cationic charge promotes prolonged interaction with the negatively charged stratum corneum. This creates a high concentration gradient that drives the miconazole through the intercellular lipid matrix of the skin. Furthermore, vesicles of ~500 nm are known to settle in skin appendages, acting as a controlled-release reservoir that targets fungi residing in deeper epidermal layers.

## 4. Conclusions

A central composite rotatable design was used to optimize miconazole-loaded sterosomes (STEs) by maximizing entrapment efficiency and zeta potential while minimizing vesicle size. The optimized formulation exhibited appropriate particle size, surface charge, and efficient drug loading. It showed enhanced *in vitro* release compared with miconazole suspension. X-ray diffraction confirmed drug encapsulation, and TEM revealed spherical vesicles without visible aggregation. After incorporation into a hydroxypropyl methylcellulose gel, the optimized formulation demonstrated improved antifungal activity and favorable histopathological findings in a rat model of cutaneous candidiasis. These findings indicate that sterosomes are a promising topical delivery system for miconazole. Further studies addressing long-term stability, dermal pharmacokinetics, irritation/toxicity, and comparative clinical performance are required before definitive conclusions regarding superiority can be made.

## Supporting information

S1 Data Supporting information. (ZIP)
