# Supplementary material for: Topical sterosomes-based nanocarrier of miconazole for the management of cutaneous candidiasis
Source: PLoS One. 2026 Jul 10;21(7):e0353060. doi: 10.1371/journal.pone.0353060 (PMC13354107; doi:10.1371/journal.pone.0353060)
Supplement: S1 Data — (ZIP) [file pone.0353060.s001.zip › supporting information/Supporting Information raw data for in vitro antifungal.docx]

**Raw Data Inhibition zone in mm in triplicate independent experiments for antifungal activity for drug and optimized formula and results reported as mean ± SD**

| Standard drug 1 | Standard drug 2 | Standard drug 3 | Mean ± SD (mm) | Optimized exper 1 | Optimized exper 2 | Optimized exper 3 | Mean ± SD (mm) |
| --- | --- | --- | --- | --- | --- | --- | --- |
| 31 | 30 | 31 | **30.67 ± 0.58** | 33 | 33 | 31 | **32.33 ± 1.15** |
| 29 | 30 | 28 | **29.00 ± 1.00** | 30 | 30 | 31 | **30.33 ± 0.58** |
| 27 | 27 | 28 | **27.33 ± 0.58** | 29 | 29 | 28 | **28.67 ± 0.58** |
| 23 | 23 | 24 | **23.33 ± 0.58** | 27 | 26 | 27 | **26.67 ± 0.58** |
| 21 | 21 | 22 | **21.33 ± 0.58** | 25 | 25 | 23 | **24.33 ± 1.15** |
| 20 | 20 | 19 | **19.67 ± 0.58** | 21 | 21 | 22 | **21.33 ± 0.58** |
| 19 | 19 | 18 | **18.67 ± 0.58** | 20 | 19 | 20 | **19.67 ± 0.58** |
| 18 | 18 | 17 | **17.67 ± 0.58** | 18 | 17 | 17 | **17.33 ± 0.58** |
| 15 | 15 | 14 | **14.67 ± 0.58** | 11 | 10 | 11 | **10.67 ± 0.58** |
| 11 | 11 | 10 | **10.67 ± 0.58** | – | – | – | **-** |
