# Supplementary figures and images for: Topical sterosomes-based nanocarrier of miconazole for the management of cutaneous candidiasis
Source: PLoS One. 2026 Jul 10;21(7):e0353060. doi: 10.1371/journal.pone.0353060 (PMC13354107; doi:10.1371/journal.pone.0353060)

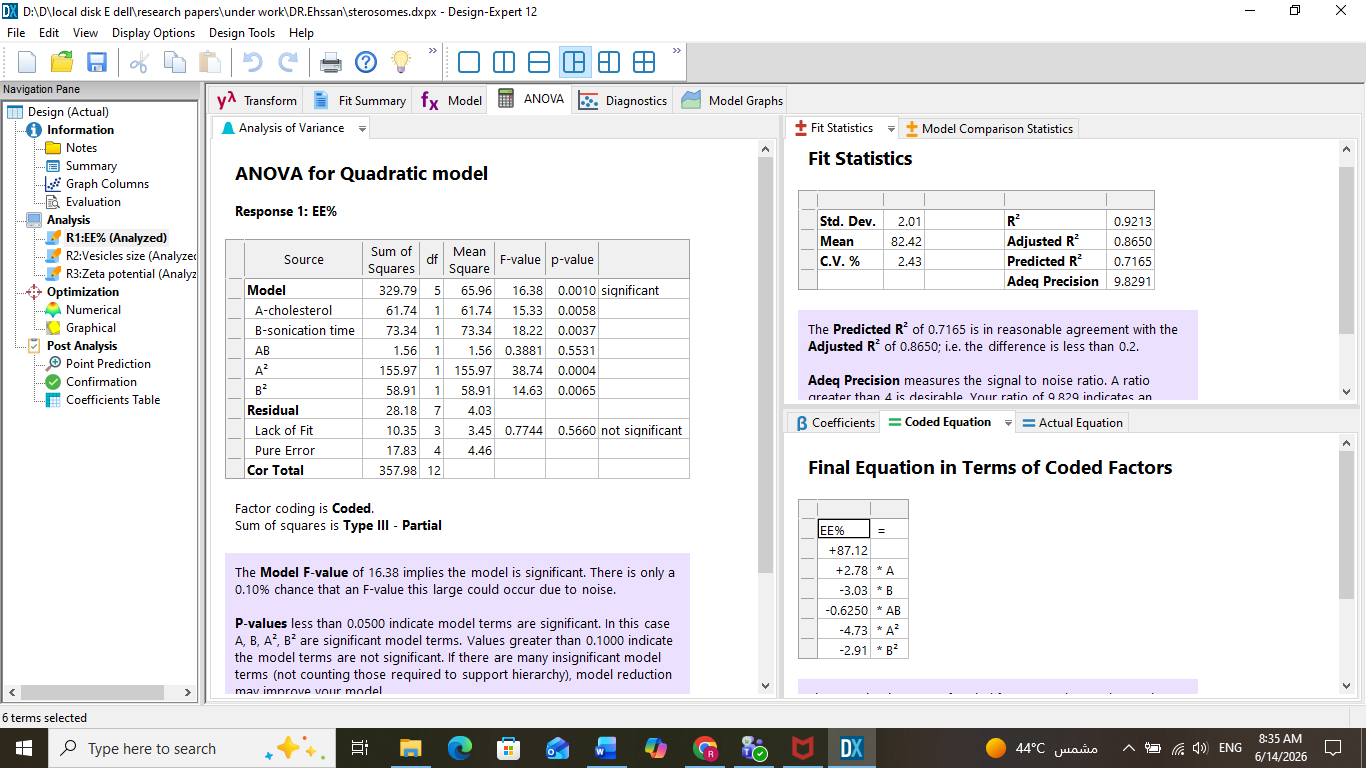


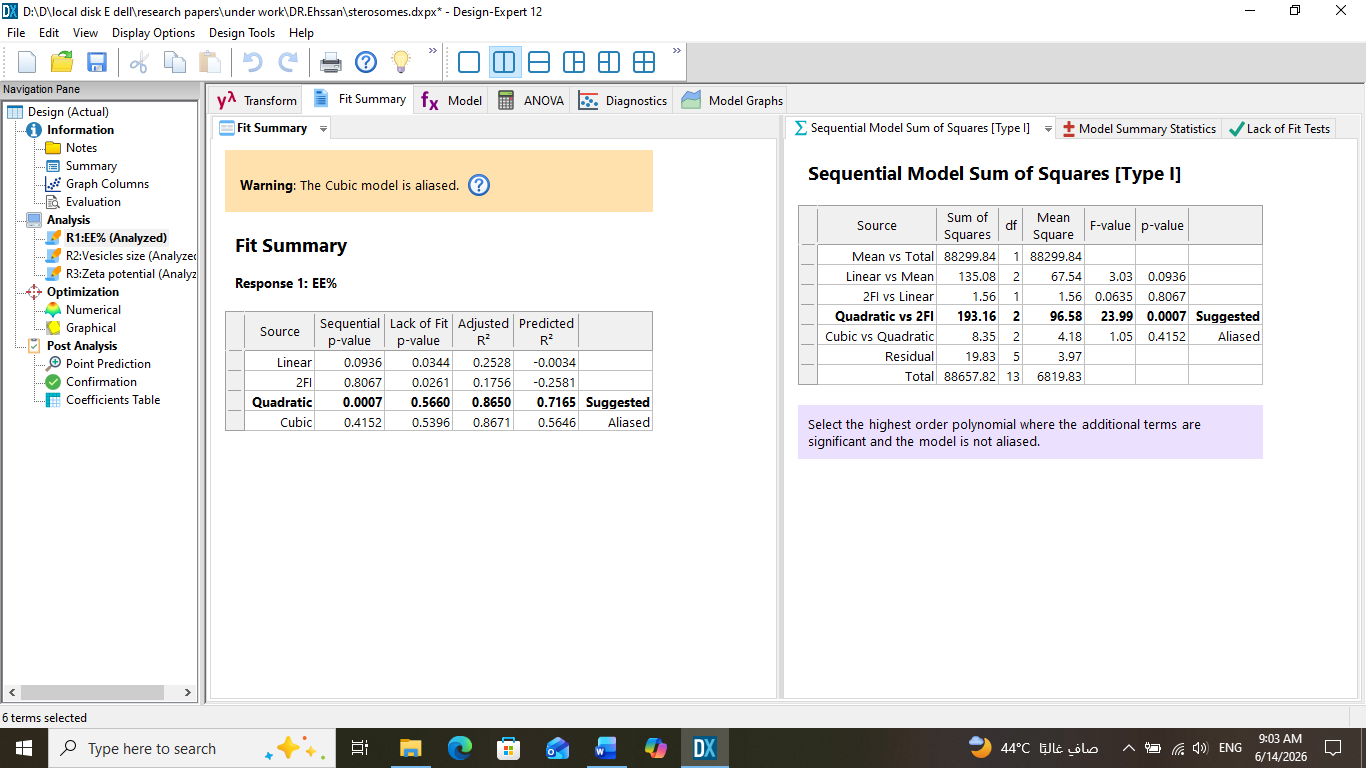

Supplement: S1 Data — (ZIP) [file pone.0353060.s001.zip › supporting information/Supporting information raw data EE%.docx]

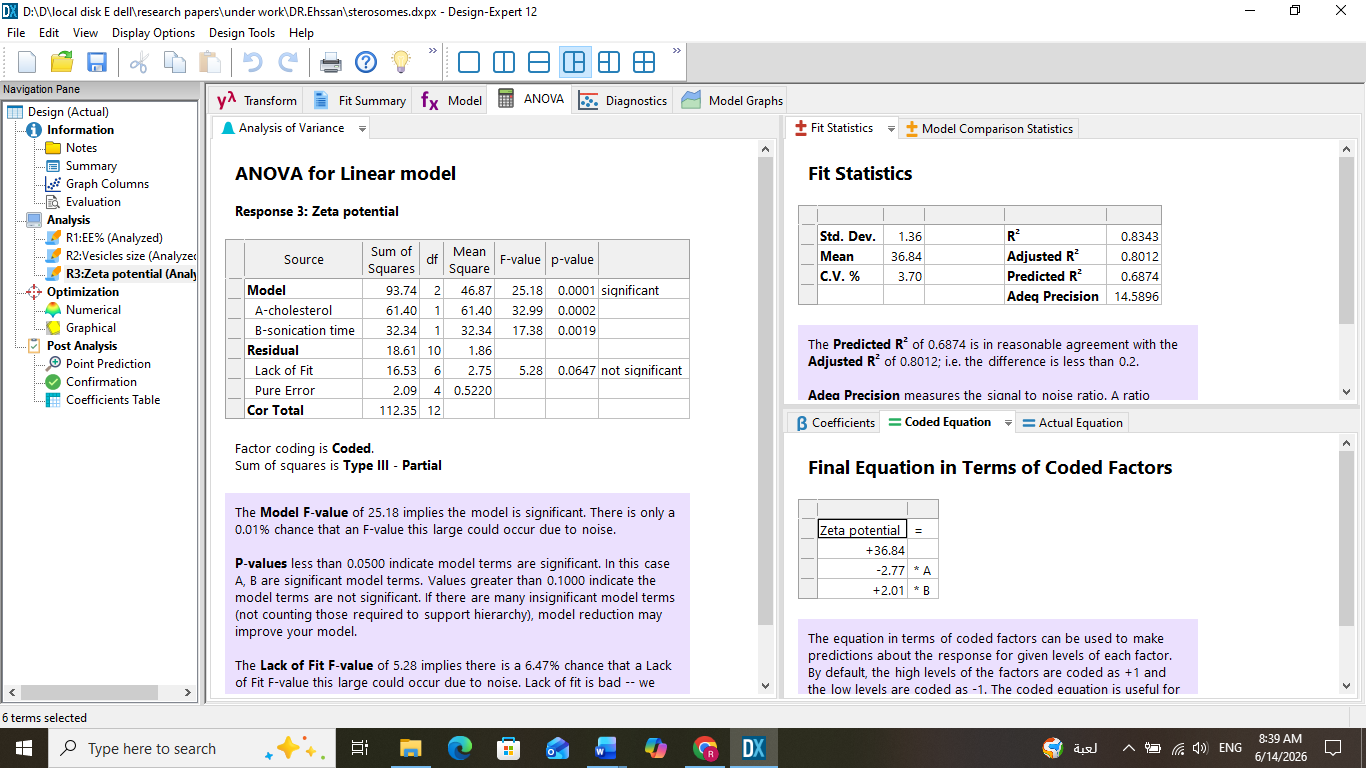


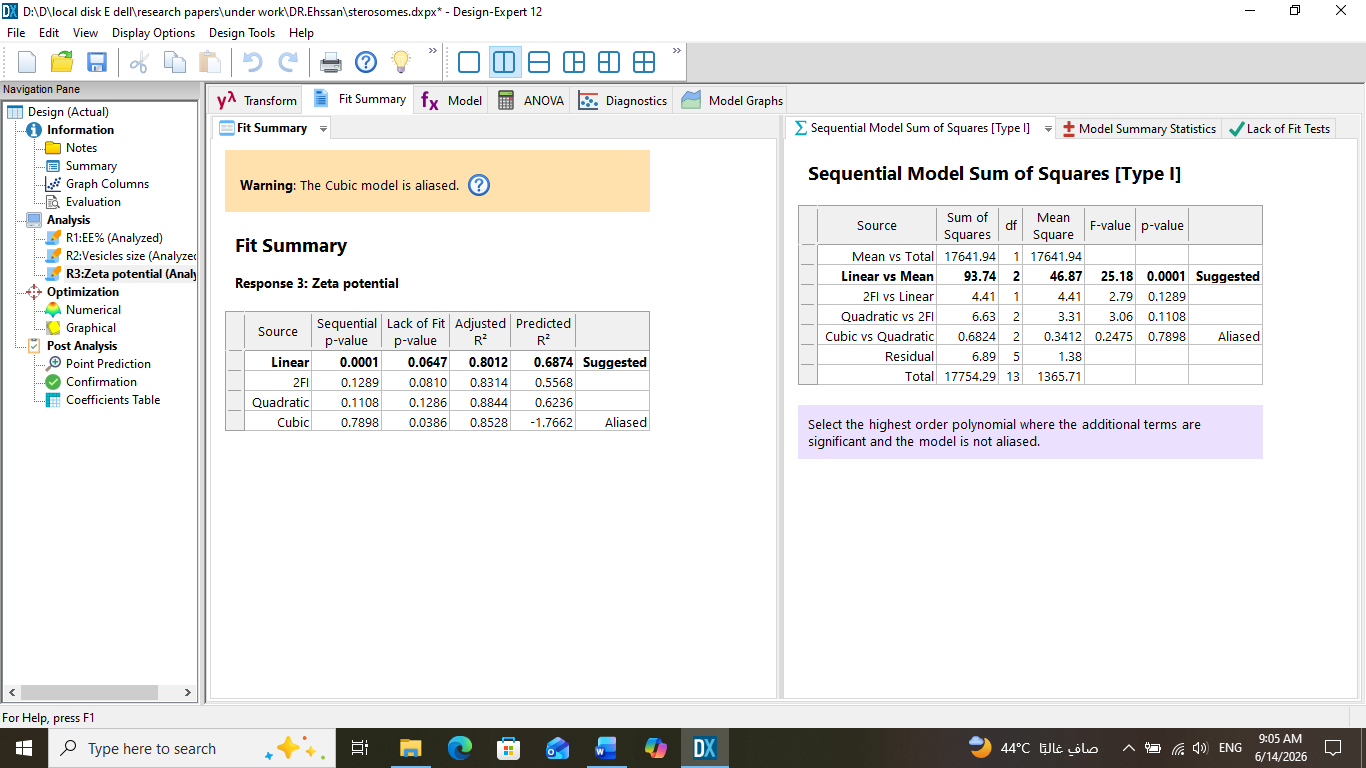

Supplement: S1 Data — (ZIP) [file pone.0353060.s001.zip › supporting information/supporting information raw data zeta potential.docx]

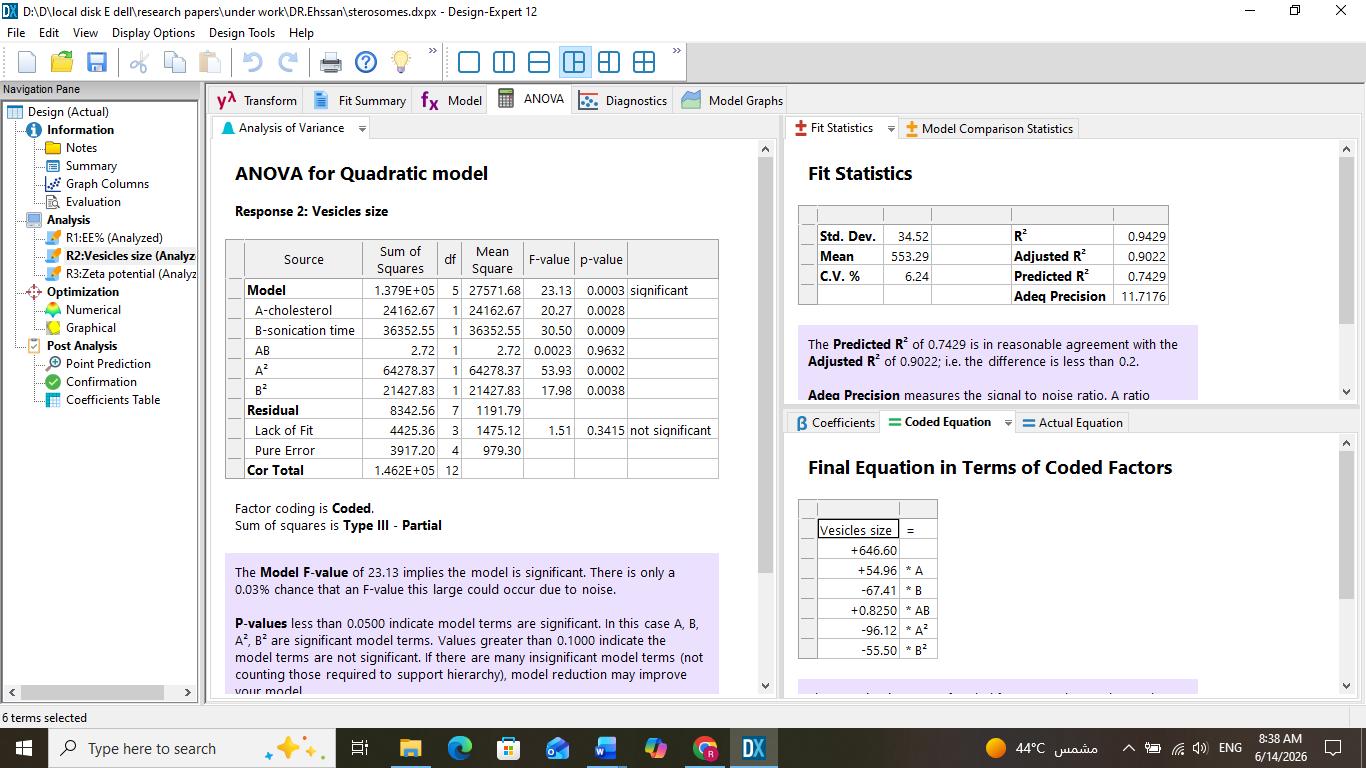


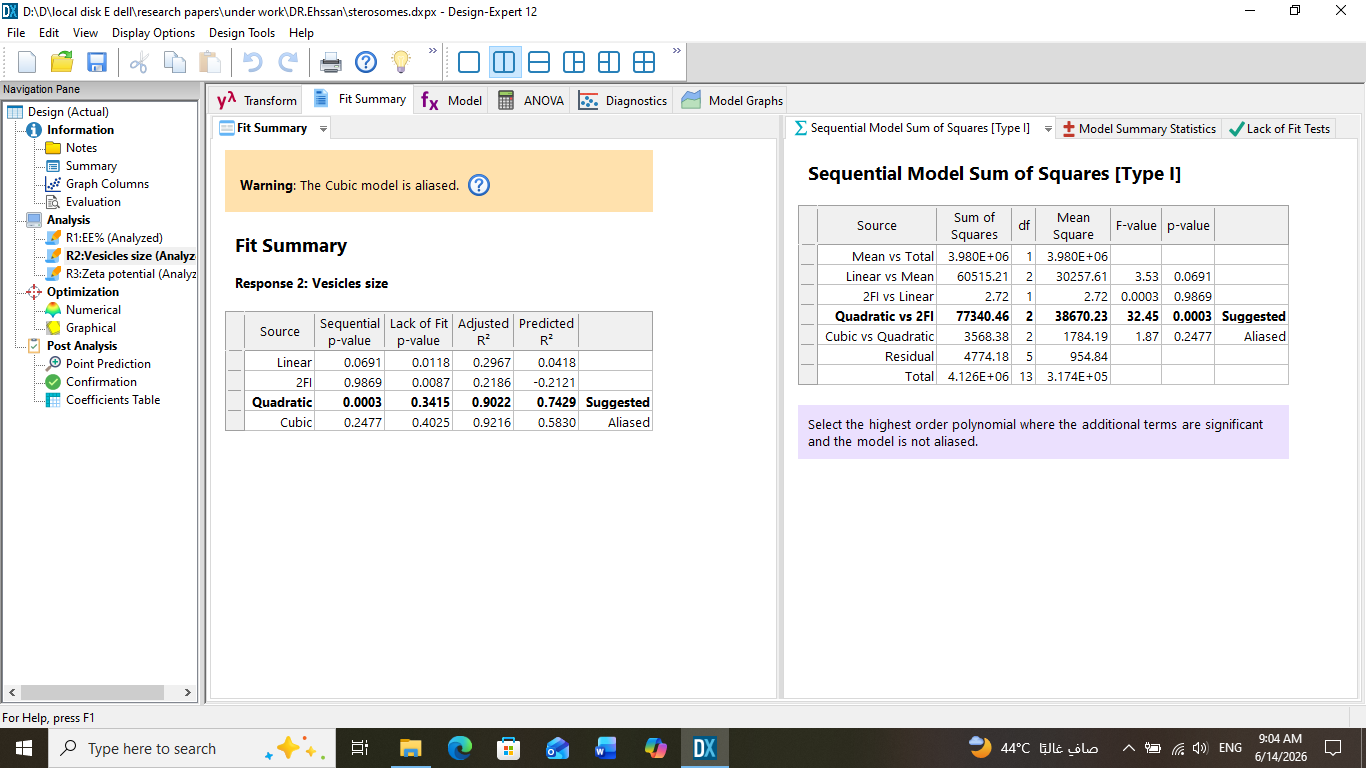

Supplement: S1 Data — (ZIP) [file pone.0353060.s001.zip › supporting information/suppoting information raw data Particle size.docx]
